# New‐onset lung sarcoidosis, an adverse event by COVID‐19 or a sign of convalescence; a case report

**DOI:** 10.1002/ccr3.7339

**Published:** 2023-05-10

**Authors:** Somayeh Sadeghi, Shadi Reisizadeh Mobarakeh, Mahnaz Momenzadeh, Amir Aria, Mitra Heidarpour, Somayeh Haji Ahmadi, Zohreh Naderi

**Affiliations:** ^1^ Acquired Immunodeficiency Research Center, Al‐Zahra Hospital Isfahan University of Medical Sciences Isfahan Iran; ^2^ Department of Internal Medicine, Alzahra Hospital Isfahan University of Medical Sciences Isfahan Iran; ^3^ Department of Clinical Pharmacy and Pharmacy Practice Isfahan University of Medical Sciences Isfahan Iran; ^4^ Department of Pathology Isfahan University of Medical Sciences Isfahan Iran; ^5^ Department of Radiology, School of Medicine Isfahan University of Medical Sciences Isfahan Iran

**Keywords:** COVID‐19, dyspnea, granuloma, lymphadenopathy, sarcoidosis

## Abstract

**Key Clinical Message:**

Sarcoidosis is a systemic inflammatory disease able to affect any organ within the body. Sarcoidosis may be the body's secondary response to COVID‐19 infection and a sign of rehabilitation. Early response to the treatments reinforces this hypothesis. The majority of sarcoidosis patients require immunosuppressive therapies, including corticosteroids.

**Abstract:**

Most studies so far have focused on the management of COVID‐19 in patients suffering from sarcoidosis. Nevertheless, the current report aims to present a COVID‐19‐induced sarcoidosis case. Sarcoidosis is a systemic inflammatory disease with granulomas. Still, its etiology is unknown. It often affects the lungs and lymph nodes. A previously healthy 47‐year‐old female was referred with the following chief complaints: atypical chest pain, dry cough, and dyspnea on exertion within a month after COVID‐19 infection. Accordingly, a chest computed tomography revealed multiple conglomerated lymphadenopathies in the thoracic inlet, mediastinum, and hila. A core‐needle biopsy from the nodes revealed non‐necrotizing granulomatous inflammation, sarcoidal type. The sarcoidosis diagnosis was proposed and confirmed by a negative purified protein derivative (PPD) test. Accordingly, prednisolone was prescribed. All symptoms were relieved. A control lung HRCT was taken 6 months later, showing the lesions had disappeared. In conclusion, sarcoidosis may be the body's secondary response to COVID‐19 infection and a sign of disease convalescence.

## 1 INTRODUCTION

The emergence of the coronavirus disease pandemic in late 2019 has changed and challenged the practice of medicine dramatically. This virus, named the severe acute respiratory syndrome coronavirus 2 (SARS CoV‐2), causes a wide range of symptoms, from asymptomatic cases to mild flu‐like conditions to severe cases with multi‐organ failure and severe respiratory distress and even death.^1^

Sarcoidosis is a systemic inflammatory disease that can affect any organ within the body; however, over 90% of the patients exhibit lung involvement, and approximately all have manifestations in more than one organ. Most sarcoidosis patients require immunosuppressive therapies, including corticosteroids.^2^

Most studies so far have focused on the management of COVID‐19 in patients suffering from sarcoidosis due to the nature of their disease and chronic treatment with agents negatively affecting their immune competence.^3^ Nevertheless, a hypothesis presenting common molecular mechanisms between COVID‐19 and sarcoidosis has been proposed. Accordingly, this paper aims to explain a case with COVID‐19 infection that developed new‐onset sarcoidosis after infection with SARS‐CoV‐2.

## 2 CASE PRESENTATION

A previously healthy 47‐year‐old female was referred to a respiratory clinic affiliated with Isfahan University of Medical Sciences with the following chief complaints: atypical chest pain, dry cough, and dyspnea on exertion since a month ago.

She mentioned taking the coronavirus polymerase chain reaction (PCR) due to the COVID‐19 pandemic and her occupation as a nurse when the symptoms presented. After the positive test, she was quarantined for about 3 weeks using no medications.

She was admitted to this clinic after her symptoms progressed. A comprehensive interview was done, and she denied any other symptoms, such as weight loss, hemoptysis, night sweating, and fever. No history of active or passive smoking was presented. Her vital signs were normal, including oral temperature, blood pressure, heart rate, respiratory rate, and pulse oximetry in room air at rest. Afterward, a complete physical examination was performed, and no significant abnormality was noted.

Due to the deteriorating dyspnea, a chest X‐ray was taken, notifying mediastinum widening. Therefore, lung high‐resolution computed tomography (HRCT) was performed.

**FIGURE 1:**
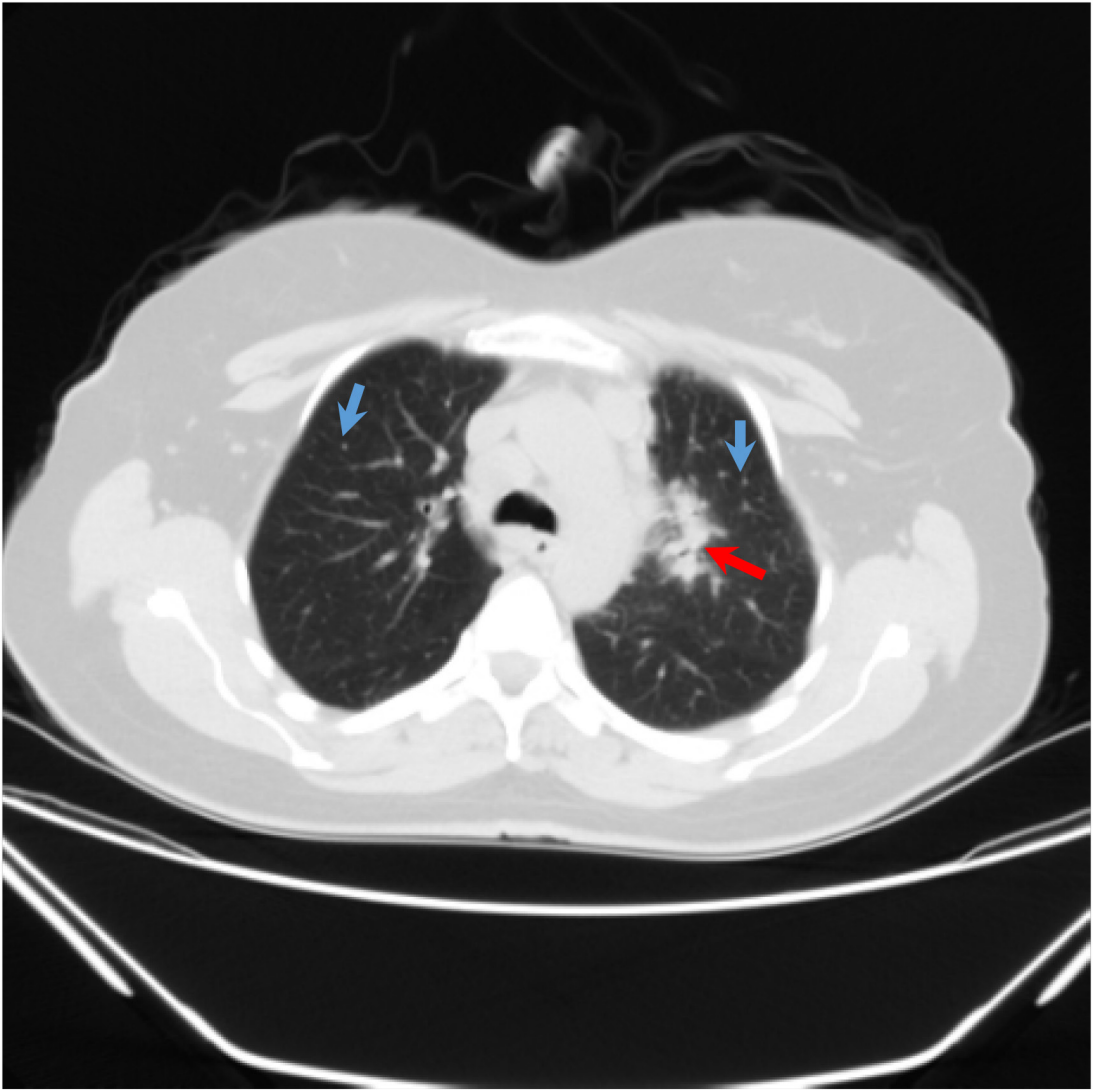
Lung HRCT, showing left hilar mass (red arrow) and bilateral nodules (blue arrows).

The HRCT interpretation revealed a left lung hilar mass with perivascular, aortopulmonary, and up to 27 mm local lymphadenopathies (LAPs) in the subcarinal region. Besides, bilateral pulmonary nodules up to 4.5 mm in the superior segment of the right lower lobe were observed. The heart size was normal. These findings were suggestive of non‐small cell lung bronchogenic carcinoma. Therefore, contrast‐enhanced CT for further evaluation was recommended. Figure 1 demonstrates the HRCT.

**FIGURE 2:**
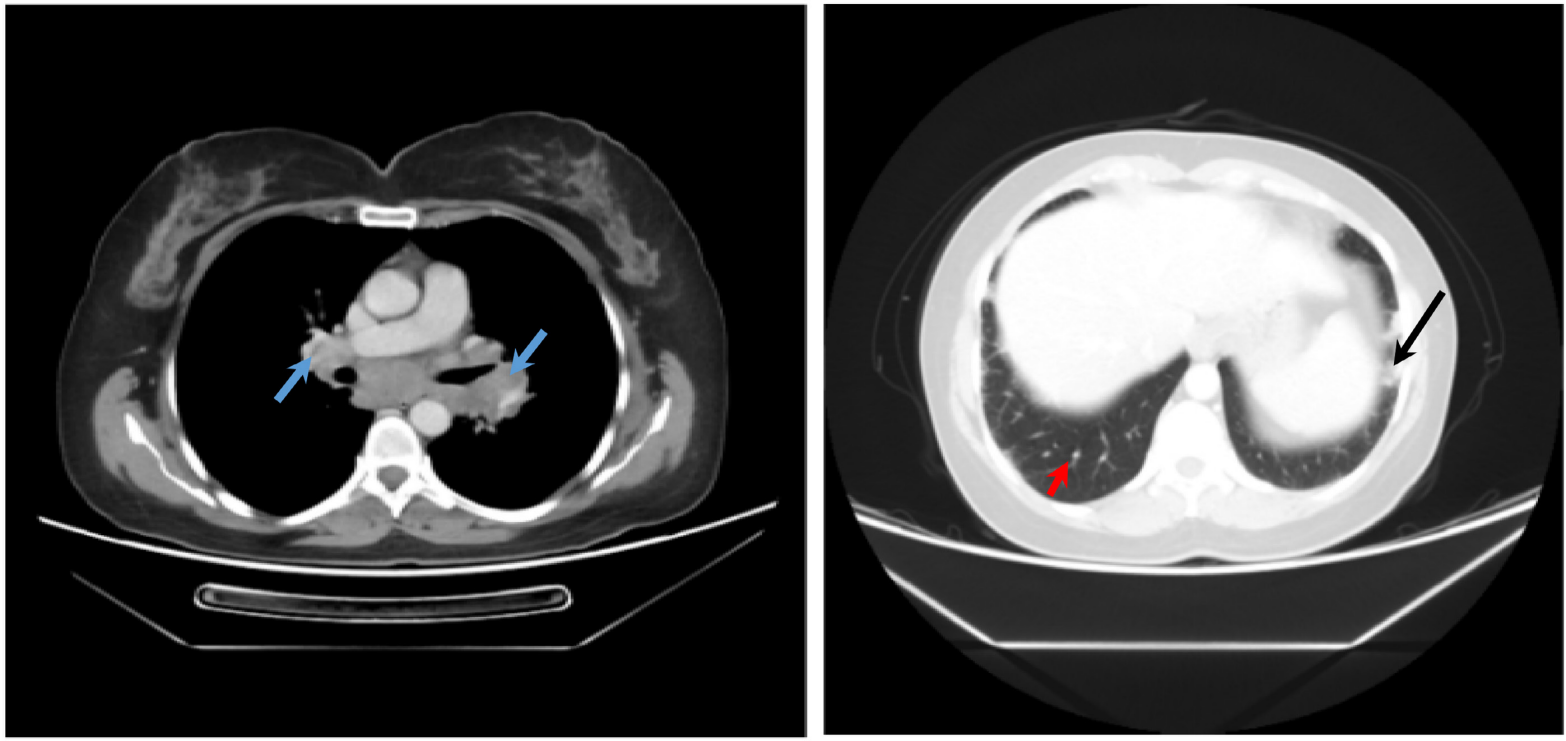
Lung MDCT showing pulmonary nodules (red arrow), mediastinal and hilar LAPs (blue arrows) with fibrotic changes (black arrow) due to COVID‐19 pneumonia.

In the next step, multidetector computed tomography (MDCT) was done. Multiple conglomerated LAPs in the thoracic inlet, mediastinum, and hila up to 27*39 mm in the subcarinal region were detected, with some being necrotic. Besides, numerous bilateral pulmonary nodules up to 8 mm in the left lower lobe and subpleural reticular densities in both lower lobes were notified. These findings were suggestive of fibrotic changes caused by the previous COVID‐19 pneumonia. Bony thorax and soft tissue were normal. There was no evidence of pleural effusion or thickening (Figure 2).

**FIGURE 3:**
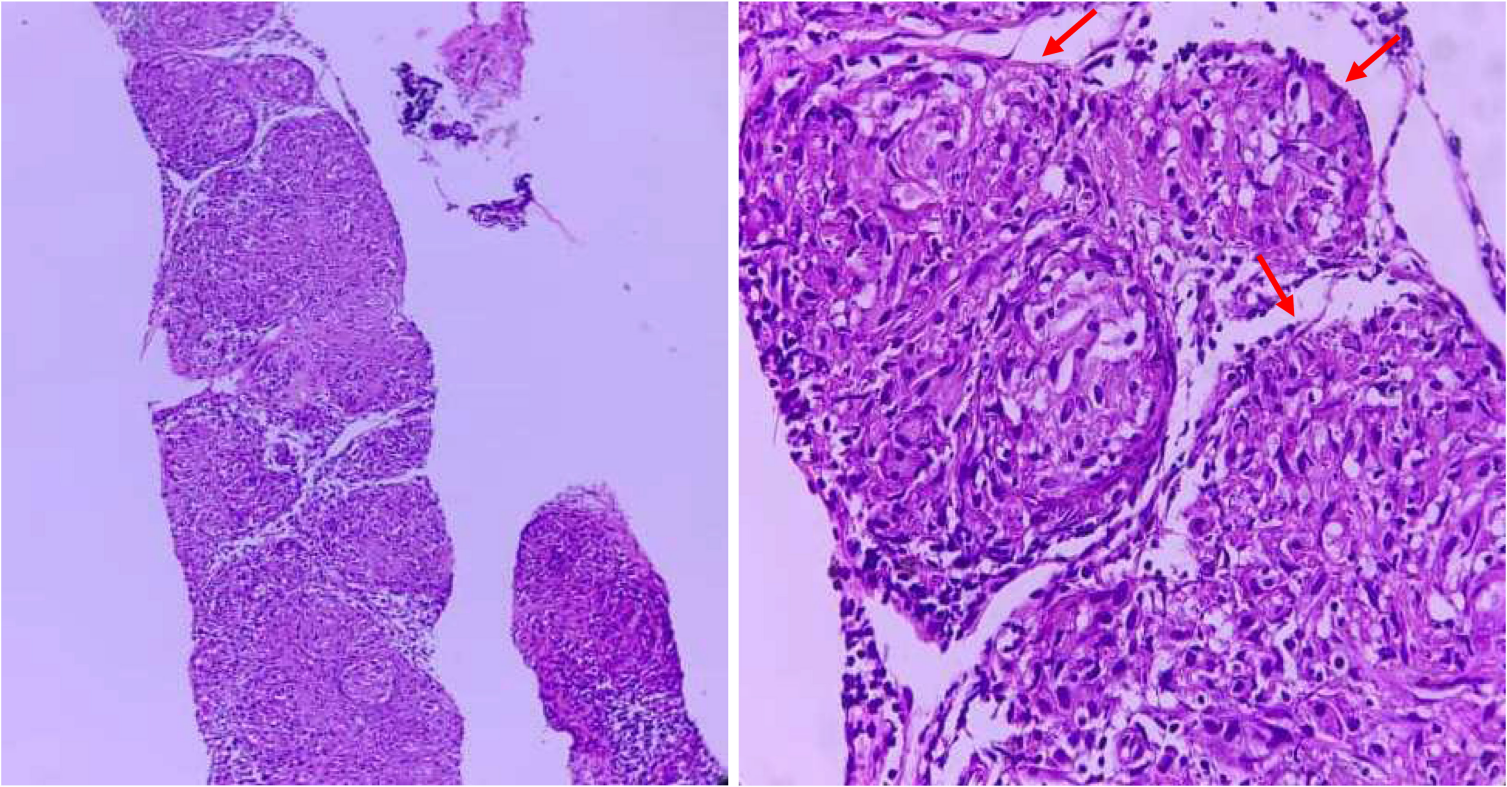
Lymph node tissue with nodal effacement by small non‐necrotizing granulomas comprising epithelioid cells with scattered Langhans giant cells and lymphocytes (red arrows).

To establish the diagnosis, a CT‐guided core‐needle biopsy from the mediastinal lymph node was taken and interpreted as follows:

The sections showed lymph node tissue with nodal effacement by small non‐necrotizing granulomas comprising epithelioid cells with scattered Langhans giant cells and lymphocytes. There is no evidence of malignancy (Figure 3).

These findings were compatible with the sarcoidosis diagnosis. Therefore, a purified protein derivative (PPD) test for tuberculosis was performed, which was negative. Further examinations of the eyes and other organs were normal.

Accordingly, 0.5 mg/kg/day of prednisolone was initially prescribed and tapered to 7.5 in 6 months. All symptoms were relieved, and she did not complain of any other problems during this period. A control lung HRCT was taken after 6 months of treatment, showing the lesions had disappeared.

## 3 DISCUSSION

To our knowledge, COVID‐19‐induced lung sarcoidosis has rarely been reported. However, some surprising cases were found too. Accordingly, a case report presented a patient with COVID‐19 pneumonia who complained of painful violaceous nodules on her skin that were biopsied and interpreted as non‐caseating granulomas suggestive of sarcoidosis. The final diagnosis was a sarcoid‐like immune reaction to SARS‐CoV‐2.^4^ The other proposed case had died due to acute respiratory distress syndrome (ARDS) following COVID‐19, whose mediastinal lymph nodes were biopsied, detecting many non‐caseous epithelial cell granulomas. Nevertheless, sarcoidosis's precedence over COVID‐19 or being a consequence of this disease remained a question.^5^

Sarcoidosis is a chronic multisystem disease characterized by the presence of non‐caseating granulomas. Although the etiology has not yet been well‐elucidated, its pathophysiology can be described as an antigen‐driven process. The granulomatous lesions result from an exaggerated immunological response in genetically susceptible individuals exposed to persisting, so far not definitively defined, environmental antigens. These antigens range from non‐infectious agents, such as beryllium, other metals, clay, and pollen, to infectious ones, such as Mycobacterium tuberculosis, nontuberculous Mycobacteria, Propionibacterium acnes, other bacteria, and several viruses, such as herpes viruses.^6^ Accordingly, it is presumable that an antigen in the structure of SARS CoV‐2 may be responsible for the incidence of sarcoidosis in the introduced case.

Since epithelial cells lining the granulomas produce ACE in sarcoidosis and sarcoid‐like reactions, high levels of ACE are noted in these patients.^7^ The expression of ACE by granulomas in sarcoidosis has been hypothesized to link COVID‐19 infection and the emergence of sarcoidosis in previously healthy cases.

There is a strong but finely regulated interaction between autophagy, programmed cell death, and apoptosis in COVID‐19. Cell entry of SARS‐CoV‐1 and ‐2 depends on viral spike (S) proteins binding to cellular receptors, such as angiotensin‐converting enzyme (ACE)‐2, expressed on mucosal and bronchial cells in humans.^8^

Due to the inverse association between the ACE‐2 receptor and ACE expression, elevated levels of ACE accompanies suppressed ACE‐2.^9^ Therefore, we postulate that in response to the invasion of SARS‐CoV‐2 viruses to the lung and consumption of ACE‐2 receptors, a downregulating cascade may get activated by which ACE production increases to restrict the ACE‐2 receptors' accessibility for more severe involvement. Therefore, a sarcoid‐like reaction and even sarcoidosis incidence is not a surprising response to this mechanism.^4^ Therefore, sarcoidosis incidence in COVID‐19 patients can be considered a sign of convalescence rather than an acute infection itself, as the symptoms initiated after a month following COVID‐19 infection in this case.

The other confirmatory assessments regarding the associative pathophysiology of sarcoidosis and COVID‐19 rely on the pathological examination of lung biopsy tissues from COVID‐19 patients, showing the presence of inflammatory clusters, including multinucleated giant cells (MGCs) and CD4+ T lymphocytes, an observation reminiscent of MGCs in sarcoidosis.^8^

The rapidly resolving nature of the lymphadenopathy and negative systemic workup further support COVID‐19‐induced sarcoidosis rather than a diagnosis of primary sarcoidosis.

## 4 CONCLUSION

In conclusion, sarcoidosis and sarcoid‐like reactions may be the body's secondary response to COVID‐19 infection and a sign of disease rehabilitation. Early treatment response reinforces this hypothesis; however, further investigations are required.

## AUTHOR CONTRIBUTIONS

**Somayeh Sadeghi:** Conceptualization; formal analysis; investigation; project administration. **Shadi Reisizadeh Mobarakeh:** Conceptualization; formal analysis; investigation. **Mahnaz Momenzadeh:** Data curation; investigation; writing – review and editing. **Amir Aria:** Investigation; resources; validation; writing – original draft; writing – review and editing. **mitra heidarpour:** Validation; writing – original draft. **Somayeh Haji Ahmadi:** Validation; writing – original draft. **zohreh naderi:** Writing – original draft.

## FUNDING INFORMATION

None.

## CONFLICT OF INTEREST STATEMENT

The authors declared no conflicts of interest in this study.

## CONSENT STATEMENT

Written informed consent was obtained from the patient to publish this report in accordance with the journal's patient consent policy.

## Data Availability

Data sharing is not applicable to this case report type article as no new data were created or analyzed in this study.
